# Mini-Invasive Thoracic Surgery for Early-Stage Lung Cancer: Which Is the Surgeon’s Best Approach for Video-Assisted Thoracic Surgery?

**DOI:** 10.3390/jcm13216447

**Published:** 2024-10-28

**Authors:** Beatrice Trabalza Marinucci, Alessandra Siciliani, Claudio Andreetti, Matteo Tiracorrendo, Fabiana Messa, Giorgia Piccioni, Giulio Maurizi, Antonio D’Andrilli, Cecilia Menna, Anna Maria Ciccone, Camilla Vanni, Giacomo Argento, Erino Angelo Rendina, Mohsen Ibrahim

**Affiliations:** Department of Thoracic Surgery, Sant’Andrea Hospital, Sapienza University, 00189 Rome, Italy; alessandrasiciliani@gmail.com (A.S.); claudio.andreetti@uniroma1.it (C.A.); tiracorrendomatteo@gmail.com (M.T.); fabiana.messa@uniroma1.it (F.M.); giorgia.piccioni@uniroma1.it (G.P.); giulio.maurizi@uniroma1.it (G.M.); adandrilli@hotmail.com (A.D.); cmrossi.85@gmail.com (C.M.); cicconeam@gmail.com (A.M.C.); camillavanni.1@gmail.com (C.V.); giacomo.argento@uniroma1.it (G.A.); erinoangelo.rendina@uniroma1.it (E.A.R.); mohsen.ibrahim@uniroma1.it (M.I.)

**Keywords:** video-assisted thoracic surgery, lung cancer, minimally invasive

## Abstract

**Objectives:** The choice of the best Video-Assisted Thoracic Surgery (VATS) surgical approach is still debated. Surgeons are often faced with the choice between innovation and self-confidence. The present study reports the experience of a high-volume single institute, comparing data of uni-portal, bi-portal and tri-portal VATS, to find out the safest and most effective mini-invasive approach, leading surgeon’s choice. **Methods:** Between 2015 and 2022, a total of 210 matched patients underwent VATS lobectomy for early-stage cancer, using uni-portal (fifth intercostal space), bi-portal (seventh space for optic and the fifth), and tri-portal (seventh and the fifth/four) access. Patients were matched for age, BPCO, smoke, comorbidities, lesions (size and staging) to obtain three homogenous groups (A: uni-portal; B: bi-portal; C: tri-portal). The surgeons had comparable expertise. Data were retrospectively collected from institutional database and analyzed. **Results:** No differences were detected considering time of surgery, length of hospital stay, complications, conversion rate, specific survival, and days of chest tube length of stay. Better results on chest tube removal were described in group A (mean 1.1 days) compared to B (mean 2.6 days) and C (mean 4.7 days); nevertheless, they not statistically significant (*p* = 0.106). **Conclusions:** No significant differences among the groups were described, except for the reduction in chest tube permanence in group A. This allows to hypothesize an enhanced recovery after surgery in this group but the different approaches in this series seem to guarantee comparable safety and effectiveness. Considering no superiority of one method above the others, the best suggested approach should be the one for which the surgeon feels more confident.

## 1. Introduction

The advantages of Video-assisted Thoracic Surgery (VATS) for an enhanced recovery after surgery are well known. In fact, as a novel mini- and minimally invasive thoracic surgery technique, VATS enables surgeons to perform surgery through smaller incisions, guaranteeing less morbidity and a faster recovery compared to open thoracotomy, reducing postoperative pain, hospital stay, and improving patient quality of life and satisfaction [1]. VATS represent one of the major progresses in the history of thoracic surgery: the development of VATS and its learning curve from multi-portal to bi-portal, to uni-portal access, has been a great advance in thoracic surgery in the last 30 years. VATS is being constantly rejuvenated by the development of the techniques, with the aim to reduce the size and/or number of accesses to reduce surgical access trauma. In fact, over the past decade, VATS with three or four ports (including a 2–4 cm utility incision for delivery of the resected lobe) has been evolved to produce bi-portal (utility mini-thoracotomy with another additional thoracoscopic port), for which the locations of incision varied according to the preference of the surgeon, and finally to uni-portal (only 1-utility mini-thoracotomy) [2]. Uni-portal minimally invasive surgery has developed rapidly since Dr. Rocco first reported it in 2013, expanding from the minor thoracic procedures, such as wedge resection, to complex operations, such as lobectomy, segmentectomy, and even bronchial or pulmonary angioplasty [3].

Bi-portal VATS imply two radical changes in perspectives from the traditional three-portal technique. For double port VATS lower lobectomies, all the instrumentation and stapler insertions are performed through the utility incision. To introduce staplers without the third conventional posterior port, the location of the endostapler and the thoracoscope have to be interchanged between the two incisions. There is another important aspect, related to the instrumentation interference. In fact, instrumentation with both proximal and distal articulation, modern articulated staplers, high definition 30° cameras and energy devices seem to be more fitted for successful biportal VATS lobectomy. Moreover, bi-manual instrumentation using a cross-hand technique is often used [4]

In recent years, uni-portal VATS (U-VATS) has become a new safe and effective technique in the surgical treatment for non-small cell lung cancer (NSCLC) [5].

The single, uni-portal VATS is an approach meant to reproduce the open technique transferring the operative fulcrum inside the chest secondary to the introduction of articulating instruments. The approach is different than conventional, three- VATS because it develops along a sagittal plane, rather than a latero-lateral one. The advantage of using the camera in coordination with the instruments is that the vision is directed to the target tissue, bringing the instruments to address the target lesion from a straight perspective and, thus, it is possible to obtain similar angle of view as for open surgery. This evolution in the approach from three ports to the single port technique required a new learning curve related to different lung exposures and learning how to coordinate the instruments and the camera with no interference during surgery. So, the innovation leading to bi-VATS consisted mainly of: removing the posterior port; placing the camera at the posterior part of utility incision for lower lobectomies; using bimanual instrumentation with curved instruments; placing the camera through utility incision and use inferior port only for stapler insertion or for instrumentation for upper lobectomies; using the inferior port only to expose the lung (camera, staplers, and instrumentation through the incision) [3]. The evolution through U-Vats consists of removing the inferior port; using vascular clips when no angle for staplers; always inserting the staplers with angulation for vascular division; adopting the Anterior small thoracotomy approach (10–12 cm incision); removing the rib spreader and moving the instruments and camera along the 10 cm incision; and finally reducing, progressively, the size of the incision after gained experience [6].

Many studies in the literature have already been focused on the feasibility and on the differences in the various VATS approaches, and several studies showed no difference between them, in terms of intra- and postoperative outcomes [7]. So, the comparative clinical outcomes of U-VATS versus multiportal VATS (M-VATS) remain uncertain, and the choice of the best mini-invasive surgical approach is still being debated, considering the pro and contra of every single approach. VATS is established today as the preferred approach for early-stage lung cancer, according to the American College of Chest Physicians (ACCP) and the European Society of Medical oncology (ESMO). Although VATS has greatly reduced surgical morbidity compared to open thoracotomy, it has not eliminated it. Studies have shown that after VATS, up to 32% of patients still experience some residual discomfort. Moreover, with the increasing spread of Robot systems, VATS is now considered one of the possible minimally invasive techniques for early-stage lung cancer. In consequence, VATS is being constantly rejuvenated by the development of the technique, aimed to reduce the size and/or number of access (three-porta, two-port, and uni-portal VATS), and there is no evidence of the superiority of one over the others. Therefore, among the different VATS approaches for NSCLC treatment, the best one has not been standardized, and the surgeon is often faced with the choice between the most innovative technique and the one that gives him the most confidence.

The objective of the present study is to report the experience of a high-volume single institute, comparing data of uni-portal, bi-portal, and tri-portal VATS, to discuss the safest and most effective mini-invasive approach, helping to lead the surgeon’s choice.

## 2. Materials and Methods

Between 2015 and 2022, a total of 240 patients underwent VATS lobectomy for early-stage lung cancer in a single center.

Inclusion criteria consisted of patients >18 years old affected by early lung cancer (stage I–IIB, excluded T3), so none underwent neoadjuvant therapy, and patients eligible for surgery using mini-invasive approach were included for upfront surgery.

Exclusion criteria included: locally advanced stages, metastatic disease, patients with low performance status not eligible for surgery, and previous thoracic and/or cardiac surgery.

To minimize selection bias, a 1:1:1 propensity score matching was performed based on predetermined confounders and baseline characteristics (age, BPCO, smoking habits, comorbidities, lesion characteristics in terms of size and staging) to identify three homogenous groups of patients. A total of 210 patients were included and divided into group A (70) that underwent uni-portal VATS; group B (70) that underwent bi-portal VATS and group C (70) that underwent tri-portal VATS. Based on the propensity score matching, 30 patients were excluded because they did not match the variables.

All patients received pre-operative assessment, including physical examination, routine blood tests, pulmonary functional tests (spirometry and blood gas analysis) and pre-operative cardiovascular tests. Imaging included Total Body Computed Tomography (CT) and Positron Emission Tomography (PET). Central tumors required bronchoscopy for endobronchial assessment and eventual diagnosis.

**Figure 1:**
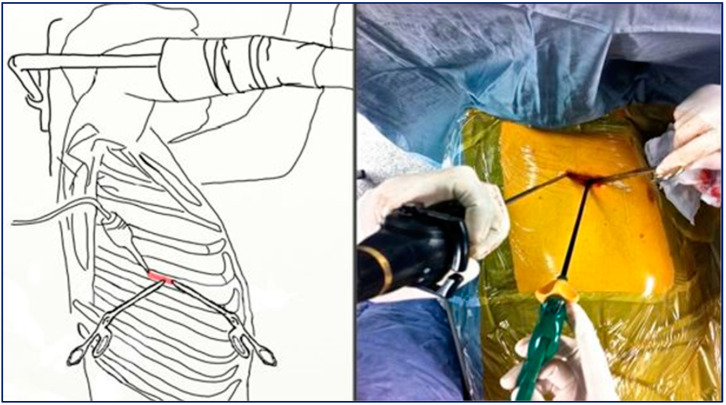
Uni-portal VATS approach.

**Figure 2:**
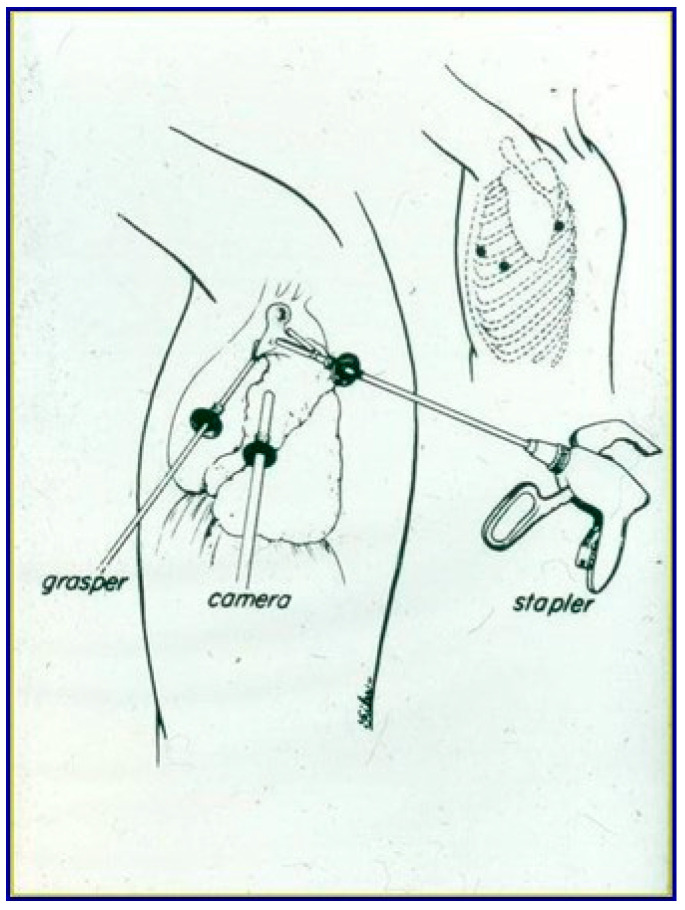
Tri-portal VATS approach.

Included patients underwent lung resection by a mini-invasive approach: uni-portal VATS (U-VATS) consisted of a single 3–4 cm instrument port [8] at either the fourth or fifth intercostal space at the anterior axillary line (Figure 1), and eventually an XS-sized Alexis wound protector (Applied Medical, Rancho Santa Margarita, CA/USA) was applied at the utility port; bi-portal VATS (BI-VATS) consisted of 1 cm camera access at the seventh–eight intercostal space, along the medium axillary line and the second 3 cm surgical access site at the fourth–fifth space at the anterior axillary line [4]; tri-portal (TRI-VATS) consisted of access of the 1 cm camera at the seventh–eight space on the mid-axillary line and the other 1 cm port instrument at the fourth–fifth intercostal space on the anterior and the posterior axillary lines (Figure 2) [9]. A 30° 10 mm thoracoscope was used for vision.

Patients received lobectomy performed in the usual manner with lymph nodes sampling. Broncho-vascular structures were sutured and divided using Endo-GIA (Covidien, Mansfield, MA, USA) or Echelon Flex (Ethicon Endo-Surgery Inc., Blue Ash, OH, USA). The resected lobe is placed inside a specimen bag before being delivered out.

As usually performed in our clinical practice, all patients received local analgesia (Ropivacaine injection in the intercostal spaces) and systemic endo-venous analgesia (Acetaminophen 1 g, three times in a day and Ketorolac 30 mg, three times in a day). Tramadol was used in case of pain persistence with the previous medicaments. Ropivacaine can have cardiotoxic side effects, and an alternative for local analgesia could be Bupivacaine. However, Ropivacaine is believed to have a lower incidence of clinical cardiac side effects than bupivacaine. The replacement of the butyl group in bupivacaine by a propyl group in ropivacaine alters its physicochemical properties. After molecular weight, the principal difference is the lower lipid solubility of ropivacaine. Physicochemical characteristics, such as lipophilicity and molecular weight, are different between ropivacaine and bupivacaine and are caused by the replacement of the butyl group by a propyl group. These appear to be significant factors that modulate potential cardiotoxic effects. Higher concentrations of bupivacaine and ropivacaine can block voltage-gated ion channels and intracellular enzyme systems, leading to reduced cardiac membrane potential and intracellular metabolism. Cardiac output may decrease because of ventricular dysrhythmias, contractile failure, or veno-vasodilation. Supraclinical concentrations of bupivacaine can result in death because of cardiovascular collapse. In fact, differences between the isomers and their physicochemical characteristics cause different binding of the isomers to the target site. This results in modulated potential cardiotoxic effects for Ropivacaine compared to Bupivacaine [10].

Chest tubes were connected to a pleur-evac device and Chest X-Rays were performed on post-operative day 1. Pulmonary re-expansion was defined complete if the lung achieved >90% of the pleural surface at chest XR; otherwise, it was defined incomplete; the rate of lung surface was calculated evaluating the “mean interpleural distance”, estimated according to the average distances between the lung and chest wall calculated at three points (apex, costophrenic sinus, midpoint) at chest XR [11].

Intra- and post-operative complications were registered. Cardiovascular post-operative complications included: atrial fibrillation (AF), hypertension, and coronary heart disease (CAD). Respiratory post-operative complications included: acute and/or chronic respiratory failure and pulmonary edema. Other complications explored in the study refer to: prolonged air leaks, intra-operative bleeding, and chronic pain.

Pain control was assessed using a Numeral Rating Scale (NRS), from 0 (no pain) to 10 (maximum level of pain) at 24 h and 72 h from surgery.

All patients started pulmonary rehabilitation programs (mobilization and respiratory exercises) on day 1. Chest tube drainage was removed after the radiological evidence of complete or near complete pulmonary expansion, absence of air leak in the pleur-evac system, and a median drain amount 5 mL/kg [12]. Pre-operative and intra-operative characteristics, post-operative minor complications (cardiovascular: atrial fibrillation; pulmonary embolism; respiratory: acute respiratory failure, pulmonary edema), pain, and specific survival were compared among the three groups.

**Figure 3:**
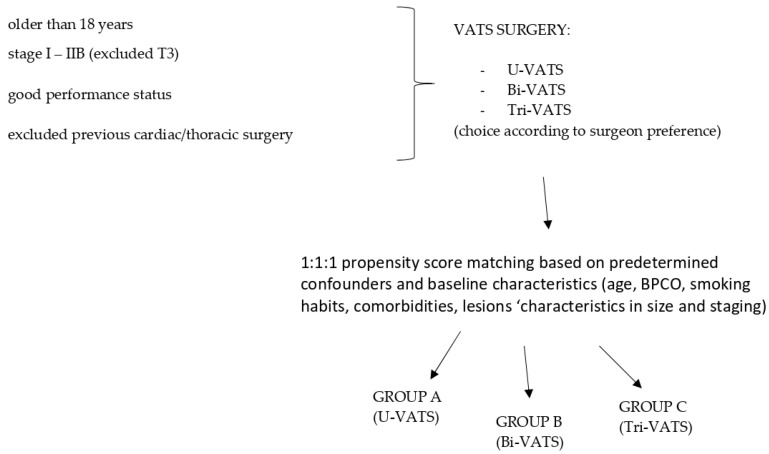
Diagram of patient recruitment.

This original retrospective observational study received institutional review board approval (Prot. n. 7 SA_2023 RIF. CE 7031/2022), and it was conducted in accordance with the Declaration of Helsinki. Written informed consent was obtained from all patients. Data are available in the text (Figure 3).

### Statistical Analysis

Data were collected and stored in an Excel database (Microsoft Corp, Redmond, WA, USA) and were analyzed using statistical package SPSS, version 25.0 (SPSS Software, IBM Corp., Armonk, NY, USA). The data collected were analyzed and compared between the three groups. Continuous variables were expressed as mean ± standard deviation (SD) and categorical variables were expressed as absolute number and percentage. The comparison of categorical variables was performed by the c2 test using Fisher’s exact test between the three groups. Comparison of continuous variables was performed by the one-way ANOVA test and post hoc Bonferroni test. Significance was defined as a *p* value of less than 0.05.

## 3. Results

General characteristics of the population are described in Table 1.

The mean age of the general population was 60.55 ± 7.1. In total, 100 patients were male (30 in group A, 30 in group B and 40 in group C, *p* = 0.826).

In total, 110 patients were smokers (50 in group A, 20 in group B, 40 in group C) without a statistically significant difference between groups (*p* = 0.263).

Cardiovascular comorbidities were referred by 30 patients in group A (42.8%), 10 in group B (14.3%), and 40 in group C (57%) (*p* = 0.243).

BPCO was detected in 40 (57%) patients in group A, 30 (42.8%) patients in group B, and 20 (28.6%) in group C (*p* = 0.558).

Stage IA3 was the most frequent, detected in 50 (71.4%) in group A, 20 (28.6%) in group B, and 40 (57%) in group C without a statistically significant difference between groups (*p* = 0.263).

The mean size of the tumor was 2.5 ± 1.2, without significant difference among the groups.

Left side lesions were described in 40 (57%) patients in group A, 40 (57%) patients in group B, and 50 (71.4%) patients in group C, without a statistically significant difference among the groups (*p* = 0.817).

LLL was performed in 20 (28.6%) patients in group A, 20 (28.6%) in group B, and 20 (28.6%) in group C, (*p* = 1.000).

LUL was performed in 20 (28.6%) patients in group A, 20 (28.6%) in group B, and 30 (42.8%) in group C, (*p* = 0.807).

RLL was performed in 20 (28.6%) in group A, 20 (28.6%) in group B, and 10 (14.3%) in group C, (*p* = 0.769).

RUL was performed in 10 (14.3%) in group A, 10 (14.3%) in group B, and 10 (14.3%) in group C (*p* = 1.000).

Surgery time was a mean of 103.20 ± 20.21, without a significant difference among the groups in the ANOVA analysis (*p* = 0.870) and in the post hoc Bonferroni test (*p* = 1.000).

The conversion rate and intra-operative massive bleeding were 0 in all the groups (*p* = 1.000).

Cardiovascular complications (atrial fibrillation-AF, hypertension, coronary heart disease-CAD) occurred in 10 patients in group A (6 AF, 3 hypertensions, 1 CAD), 0 patients in group B, and 20 patients in group C (7 AF, 12 hypertensions, 1 CAD, (*p* = 0.311).

Respiratory complications occurred in 10 (14.3%) patients in group A, 10 (14.3%) in group B, and 10 (14.3%) in group C (*p* = 1.000).

The pain score was a mean of 4.3 ± 0.5 at 24 h and 3.2 ± 1.7 at 72 h, without a significant difference among the groups.

PAL was detected in 0 patients in all groups (*p* = 1.000). Subcutaneous emphysema was described in 30 (42.8%) patients in group A, 30 (42.8%) in group B, and 20 (28.6%) in group C, (*p* = 0.807).

Incomplete pulmonary expansion in the first post-operative day was detected in 20 (28.6%) patients in group A, 10 (14.3%) in group B, and 30 (42.8%) in group C, without a statistically significant difference among the groups (*p* = 0.497).

The days of chest tube permanence were 1.14 ± 2.5 in group A, 2.6 ± 2.1 in group B, and 4.72 ± 1.5 in group C, which were not statistically significantly different according to the ANOVA test (*p* = 0.106) (Table 2) and the Bonferroni test (Table 3), considering the difference between group A and C (*p* = 0.114).

The mean total hospital stay was 4.5 ± 3.8, without a statistically significant difference among the groups according to the ANOVA test (*p* = 0.172), shown in Table 2, and the Bonferroni test (Table 3).

Peri-operative mortality was 0 in all groups.

At 1 year of follow-up, we registered no mortality nor recurrence in all the groups.

## 4. Discussion

Vats first appeared in 1990 [13], and in 1993 a VATS study group was formed from multiple institutions, analyzing more than 1700 cases [14].

Many studies have already reported the advantages of VATS compared to thoracotomy, such as lower morbidity, reduced postoperative pain, better cosmetic results, and better quality of life [15]. Conventionally, the traditional VATS, known as multiportal VATS (M-VATS), was performed through 3 or 4 small accesses. M-VATS has proven to offer reliable safety and feasibility, and it was introduced as a standard procedure for surgical treatment of early-staged NSCLC [16].

With the increasing technological advances relating to thoracic surgery, VATS lobectomy has evolved from tri-portal VATS (TRI-VATS) to bi-portal (BI-PORTAL) and finally to uni-portal (U-VATS) approach.

McKenna et al. published in 2006 the largest tri-portal VATS lobectomies series [17]. A few years later, the procedure was refined to bi-portal, with only one working port for thoracoscopy and a second for utility minithoracotomy access. D’Amico et al. described one of the largest bi-portal VATS series, including more than 600 cases of VATS lobectomy and segmentectomies [18]. Yamamoto et al. [19] and Rocco et al. in 2013 [3] were the first to introduce uni-portal VATS for pleural biopsy or minor lung resections, such as wedge resections. Finally, Gonzalez-Rivas et al. [20] described a series of major lung resection (including lobectomy, segmentary resections, and even complex surgical procedures) using the uni-portal VATS approach.

In the beginning, evolution from multi-portal to bi-portal, then to uni-portal VATS seemed to reduce unnecessary working ports; however, there are several major differences such as operation field perspective (U-VATS simulating the vision of open approach as the one of the most positive aspects of this procedure, facilitating even the operative approach for more technically- demanding procedures). However, the main limitation of U-VATS is reported to be the limited flexibility of instrument circulation; in fact, for M-VATS and BI-VATS, the camera and instruments can be placed among the utility thoracotomy and ports, while in U-VATS the intense jamming and interference of instruments is inevitable [21].

Recently, several meta-analyses have compared the peri-operative outcomes of U-VATS and M-VATS [22,23]. Some Authors have demonstrated several potential advantages of U-VATS over the others approaches, such as less intraoperative blood loss, shorter hospital stay, and reduced postoperative pain [3,24], but the results of these studies were highly heterogeneous. In fact, Lin et al. indicated that U-VATS significantly increased operation time compared to M-VATS [25]. A study by Mu et al. reported a shorter average hospital stay with U-VATS [26], while in the study of Al-Ameri et al. it was longer [27].

Some studies (Yang X.Y. et al.; Yang Z. et al.) reported that patients in U-VATS group had a significant reduction regarding blood loss, length of stay, and pain [28,29], but other studies [30] demonstrated that there was no significant difference between the U-VATS and M-VATS approach in terms of length of postoperative stay.

However, many of these meta-analyses presented some limitations. In fact, most of them were focused on benign disease, including patients who underwent wedge resections but not the surgery for NSCLC, requiring radical resection of the primary lesion and lymph node dissection, which requires anatomical pulmonary resection (segmentectomy or lobectomy).

Han et al. demonstrated that there was no significant difference between a single-incision group, two-incision group, and three-incision group in both recurrence-free survival and overall survival [30]. U-VATS might have some potential advantages over M-VATS in reducing postoperative pain and drainage duration, even though these advantages are not significant.

At the end, the previous results reported in the literature showed that U-VATS is as safe, feasible, and effective method as BI- and TRI-VATS in major lung resection, indicating that there is no significant difference in peri-operative outcomes among the different approaches [31].

The absence of cardiovascular complications in Group B is just an occasional finding, probably due to the selection of patients obtained by the matching analysis, and the result is not related to the specific type of approach. The absence of cardiovascular complications in this set of patients is probably because such complications did not occur in those patients selected by the matching analysis, so it is just an occasional finding. Moreover, the comparative analysis showed no statistical difference among the groups.

The higher incomplete expansion in the TRI-VATS group could be related to air accumulation in the subcutis, which could leak into the pleural cavity more easily with triportal access than bi-portal access. However, the difference is not diriment and not statistically significant, and finally, prolonged air leak (air leak lasting after the first 5 post-operative days) was null in all the groups.

Even in the present study, with the limit of a single institution retrospective analysis, no differences were detected regarding the time of surgery, length of hospital stay, complications, conversion rate, specific survival, and days of chest tube length of stay. Better results regarding chest tube removal were described in group A (mean 1.1 days) compared to group B (mean 2.6 days) and C (mean 4.7 days); nevertheless, this was not statistically significant (*p* = 0.106). This result allows us to hypothesize an enhanced recovery after surgery in this set of patients but, with the limits of results confined to peri-operative outcomes and a brief long-term follow-up. The three surgical approaches in this series seem to guarantee comparable results in terms of safety, effectiveness, oncological results, and patient satisfaction. Many studies in the literature have already been focused on the feasibility and on the differences in the various VATS approaches, and several studies showed no difference between them in terms of intra- and post-operative outcomes. So, the comparative clinical outcomes of U-VATS versus multi-port VATS (M-VATS) remain uncertain, and the choice of the best mini-invasive surgical approach is still being debated, considering the pro and contra of every single approach. The majority of constituent papers included in the meta-analysis were retrospective series or simpler case–control studies. The heterogeneity of the studies on this topic makes it difficult to achieve a standardized technique. Another effect of heterogeneity of these studies is the different surgeon experience and skills in the different centers. However, all these studies deal with safety and effectiveness of the different approaches. Considering the morbidity, mortality, and QoL, with particular attention towards the safety of VATS hilar dissection, the adequacy of oncological clearance, the long-term advantages over open surgery, and the relatively high costs of instruments and consumables, the efficacy regarding the treatment of the lung cancer was measured according to completeness of resection and survival.

The originality of our study is the comparison of the techniques with the specific aim of helping the surgeon in their choice of the correct approach for the patient, highlighting the importance of a personalized medicine based not only on the technical innovation but above all, on the operator’s best confidence to achieve the best result for the patient. Thus, a single-institution experience reinforces the comparability of the techniques, taking into consideration the equivalent expertise of the whole surgical équipe. Thus, there are no differences subjected to bias related to different operator skills. In conclusion, considering the equivalence of the team and procedures, our original work aims to support the surgeon’s choice in the approach to VATS.

## 5. Conclusions

In conclusion, considering no superiority of one method above the others, the best suggested approach should be the one for which the surgeon feels more confident. Less accesses are not synonymous with less complications, and self-confidence and personal safety for surgeons give the most effective result for patients.

In fact, innovation of surgical approaches is of great importance, but minimizing the size and number of incisions is only one part of minimally invasive surgery, which should always aim to preserve function, prolonging survival, and improving quality of life [27].

In conclusion, self-confidence and personal safety for the surgeon guarantee the best result for the patient, and less incisions should not to always be considered synonymous with less complications.

## Figures and Tables

**Table 1:** General characteristics of the population.

	UVATS	BIVATS	TRIVATS	*p*
Smoke, *n* (%)	50 (71.4%)	20 (28.6%)	40 (57%)	0.263
Cardiovascular comorbidity, *n* (%)	30 (42.8%)	10 (14.3%)	40 (57%)	0.243
BPCO, *n* (%)	40 (57%)	30 (42.8%)	20 (28.6%)	0.558
Conversion rate, *n* (%)	0	0	0	1.000
Intra-operative massive bleeding, *n* (%)	0	0	0	1.000
Post-op. Cardiovascular Complications, *n* (%)	10 (14.3%)	0	20 (28.6%)	0.311
Post-op Respiratory Complications, *n* (%)	10 (14.3%)	10 (14.3%)	10 (14.3%)	1.000
Prolonged Air Leak, *n* (%)	0	0	0	1.000
Incomplete pulmonary expansion in post-op day 1, *n* (%)	20 (28.6%)	10 (14.3%)	30 (42.8%)	0.497
Subcutaneous enphisema, *n* (%)	30 (42.8%)	20 (28.6%)	20 (28.6%)	0.807
Pain at 24 h, *n* (%)	3 (4.3%)	4 (5.7%)	4 (5.7%)	1.000

**Table 2:** ANOVA test.

	Sum of Squares	gl	Mean Square	F	*p*
Time of surgery	402.381	2	201.190	0.141	0.870
Days of chest tube	45.238	2	22.619	2.545	0.106
Length of stay	20.095	2	10.048	1.942	0.172

**Table 3:** Bonferroni test.

Variable	Surgical Type (i)	Surgical Type (ii)	Mean Difference	SD	*p*
Time of surgery (mean 103.20)	UVATS	BIVATS	5.714	20.206	1.000
		TRIVATS	−5.000		
	BIVATS	UVATS	−5.714	20.206	1.000
		TRIVATS	−10.714		
	TRIVATS	UVATS	5.000	20.206	1.000
		BIVATS	−1.429		
Days of chest tube	UVATS	BIVATS	−1.429	1.594	1.000
		TRIVATS	−3.571		0.114
	BIVATS	UVATS	−1.429	1.594	1.000
		TRIVATS	−2.143		0.586
	TRIVATS	UVATS	3.571	1.594	0.114
		BIVATS	2.143		0.586
Length of stay	UVATS	BIVATS	2.000	1.216	0.352
		TRIVATS	−0.143		1.000
	BIVATS	UVATS	−2.000	1.216	0.352
		TRIVATS	−2.143		0.285
	TRIVATS	UVATS	0.143	1.216	1.000
		BIVATS	2.143		0.285

## Data Availability

The original contributions presented in this study are included in this article; further inquiries can be directed to the corresponding author.
